# Progress in General Surgery

**Published:** 1901-04-27

**Authors:** 


### Progress in General Surgery.

**Antiseptics.**—As an historic record of his early researches, which led up to the antiseptic system of surgery, every reader of The Hospital will be careful to preserve Lord Lister's recent Huxley lecture.[1] The picture that he drew of himself as a student over forty years ago, puzzling over what he observed in a case of pyæmia, and seeking after an explanation of the process of inflammation, will ever be memorable. In his experiments on suppuration of wounds he incidentally discovered many interesting facts and problems which still remain unsolved. But the final results of his labours, which were inspired by Pasteur's observations, will endure in history. The vast benefits which have resulted from his untiring researches and scientific methods will always remain as one of the greatest advances of surgery during the past century, however much the manner of carrying out his principles may alter in the future. Sarwey[2] has recently shown by his experiments, reported to the German Surgical Congress, that many of the methods now employed for disinfecting the hands before surgical operations are very defective. He finds that the methods employed in making the bacteriological tests have hitherto been defective in their technique. The conclusion he draws appears to be that alcohol and soap with hot water are capable of considerably reducing the number of the pathogenic germs which exist on the hands, but that as yet no plan of ensuring absolute sterilisation has been devised. Kronig and Blumberg[3] have obtained the best results in sterilising the hands by employing a new mercurial salt—äthyl-endiamin. This salt can be used in a 1 per cent. solution upon the hands without producing irritation. This is a very valuable property, and makes this salt especially useful where there is marked infection. They employ it in their clinical work in a 1 to 300 solution, and have been extremely gratified with the results. The best way, according to König[4] to secure asepsis of an operation wound lies, not in the sterilisation of the hands nor in the use of gloves, but in a persistent effort to avoid touching the wound with the fingers at all. He has found it possible to perform joint resections, osteoplastic operations, and, in fact, most of the more common surgical procedures on the extremities, without touching the tissues otherwise than with instruments. However, the abdominal cavity is a field where digital manipulation will always be necessary, but even here, with practice, much may be accomplished, he thinks, without actual contact. An abundance of especially long and variously shaped retractors, forceps, &c. are required. Amyloform—a chemical combination between formaldehyde and starch—has recently[5] been introduced as a non-irritating antiseptic dusting powder. It is without taste or smell, and insoluble, but the fluids of the body bring about its decomposition. In wounds it gradually breaks up into its constituent parts and liberates both starch and formaldehyde. The formaldehyde, while retaining its powerful bactericidal properties, no longer gives rise to the pain so much complained of under ordinary circumstances. The chief advantages of amyloform appears to be its powers of cleansing unhealthy surfaces, the rapidity with which it checks morbid discharges, and its activity in promoting healthy granulations. Unlike iodoform and similar remedies, it does not give rise to unpleasant symptoms from absorption, and never causes pain locally.

[1] Brit. Med. Jour., Oct. 9, 1900, p. 969. [2] Amer. Jour. of Med. Sciences, Dec. 1900, p. 709; and Central. für Chirurg., 1900, No. 28. [3] Ibid. [4] Med. Rec., N.Y., Oct. 13, 1900, p. 575. [5] Pamphlet, L.W. Gans., Frankfort, 1900.

